# Determinants and effects or consequences of internal HIV-related stigma among people living with HIV in Morocco

**DOI:** 10.1186/s12889-021-10204-1

**Published:** 2021-01-19

**Authors:** Amal Ben Moussa, Rosemary M. Delabre, Virginie Villes, Mohammed Elkhammas, Aziza Bennani, Lahoucine Ouarsas, Hind Filali, Kamal Alami, Mehdi Karkouri, Daniela Rojas Castro

**Affiliations:** 1 Association de Lutte Contre le Sida, Casablanca, Morocco; 2 Community-based Research Laboratory, Coalition PLUS, Pantin, France; 3 Programme National de Lutte Contre le Sida, Ministère de la Santé, Rabat, Morocco; 4 Ecole Nationale de Santé Publique, Rabat, Morocco; 5 UNAIDS, Rabat, Morocco; 6 Aix Marseille Univ, INSERM, IRD, SESSTIM, Sciences Economiques & Sociales de la Santé & Traitement de l’Information Médicale, Marseille, France

**Keywords:** HIV, Stigma, Vulnerable populations, Healthcare disparities, Morocco

## Abstract

**Background:**

HIV-related stigma and discrimination constitute a barrier to different intervention programs. Unlike external stigma, internal stigma is not well explored in in the Middle East and North African countries, while grasping this particular form of stigma is essential to limit its effects. The present study aims to measure internal stigma effects and to identify factors associated with this kind of stigma not yet documented among people living with HIV (PLHIV) in Morocco.

**Methods:**

The PLHIV Stigma Index questionnaire (adapted and translated into French and Moroccan Arabic dialect “darija”) was used to collect information regarding the stigma and discrimination experienced by PLHIV across 8 cities in Morocco (September–October 2016). A randomly drawn cluster of 10 PLHIV, consisting of 5 men and 5 women, was drawn at each participating medical care center to achieve a nationally representative sample of PLHIV. Fifteen interviewers living with HIV and five supervisors were selected and trained to administer the questionnaire. An internal stigma score (range: 0–7), was calculated based on seven negative feelings/ beliefs. Negative binomial regression was used to identify characteristics associated with the internal stigma score.

**Results:**

Among 626 PLHIV, internal stigma was reported by 88.2%. The median [IQR] internal stigma score was 4 [2–5]. Regarding internal stigma, 51% avoided going to the local clinic when needed and 44% chose not to attend social gatherings. Belonging to at least one key population (aIRR [95%CI] = 1.15 [1.03; 1.28]), experiencing discriminatory reactions from family following HIV status disclosure (1.28 [1.11; 1.49]), avoiding HIV services for fear of stigmatization by staff (1.16 [1.05; 1.28]) and being denied health services because of HIV status (1.16 [1.03;1.32])**,** are among the factors significantly associated with an increase of the internal stigma score.

**Conclusions:**

Internal stigma is high among Moroccan PLHIV and significantly impacting their life decisions and their healthcare access. Multi-level interventions are needed to address internal stigma experienced by PLHIV in Morocco.

**Supplementary Information:**

The online version contains supplementary material available at 10.1186/s12889-021-10204-1.

## Background

HIV-related stigma and discrimination permeates at all levels of society to the point that it is widely acknowledged these factors must be addressed to have an effective and sustainable response to the HIV epidemic [1, 2]. It becomes particularly evident, and challenging, in certain parts of the world where individuals experience stigma and discrimination, criminalization and/or penalization for certain behaviors that may be considered both socially or culturally unacceptable and may increase exposure to HIV. In the Middle East and North African (MENA) region, for example, a 2011 report found that half of the countries had laws that acted as barriers to HIV-related prevention, treatment and care [3]. Indeed, these laws penalize, and/or criminalize key populations and their behaviours and practices such as homosexuality and sex work. These laws are inspired mainly by cultural and religious beliefs which consider premarital sex and same-sex as sinful behaviours and are subject to the death penalty in some MENA countries [4]. According to these religious beliefs, faith and religious practice are the best ways to safeguard populations against HIV infection. Although male circumcision, which is widely practiced in these countries, is a well-demonstrated biological mechanism to reduce HIV infections, adopting a strategy based on religious principles have shown its limits as the MENA region is the region that has seen the highest rise in number of new infections since 2001 [5].

HIV epidemiological data from the MENA region must be considered within its socio-economic and political setting. This geographic region is comprised of economically diverse countries, 10% of the global population between 15 and 49 years old, and a chaotic social and political environment which has had severe impacts on efforts to address public health issues such as HIV [6, 7]. Although HIV prevalence is low in the general population (0.1%), new HIV infections have increased by 12% since 2010, AIDS-related mortality has increased by 11% since 2010, and the percentage of people living with HIV (PLHIV) accessing antiretroviral treatment remains severely inadequate at 32% [8, 9]. According to 2017 data, new HIV infections are largely concentrated among clients of sex workers and sexual partners of individuals belonging to other key populations, sex workers, men who have sex with men (MSM), and people who inject drugs [8]. A review published in 2016 on trends in HIV epidemiology in the MENA region highlighted the difficulty of obtaining reliable information on transmission routes due to inadequate surveillance systems and, in particular, cultural and social norms and policies that marginalize, penalize, and/or criminalize behaviors which may lead to lack of disclosure of transmission routes [6].

Morocco is among a few number of countries in the region that have initiated a strong response to the HIV epidemic in the last decades. Morocco has an estimated 21,000 PLHIV and HIV prevalence in the general population is estimated to be < 0.1% [10]; 67% of new HIV infections occurs among key populations and their sexual partners. The bolstering of the national surveillance system [11], development of national harm reduction strategies [8], implementation of community-based education interventions and testing [12] and harm reduction advocacy and services [13], advocacy for rights for PLHIV [14] and most recently a pilot PrEP project [15] have all contributed to the national HIV response. Despite these advances, key populations in Morocco and elsewhere continue to disproportionately bear the burden of HIV and experience stigma and discrimination [16–18].

HIV-related stigma and its deleterious effects on the overall well-being of PLHIV as well as their access to testing and treatment has been well documented since the beginning of the epidemic [19–22]. Adding further complexity is the acknowledgement that PLHIV may engage in behaviors that are regarded as unacceptable in mainstream society such as injection drug use and sex work, resulting in “layered stigma” [20] or “intersectional stigma” [21]. Some authors, however, suggest that the difficulty of “defining, measuring, assessing impact of, and reducing stigma” may be the reasons behind the failure or lack of effective actions [22]. Stigma, which has been defined as an “attribute that makes him [an individual] different from others in the category of persons available for him to be, and of a less desirable kind” [23], has been categorized into two forms [24]: “external” or “enacted” stigma which refers to the experience of discrimination and “internal” or “felt” stigma refers to the feelings of blame or guilt associated with HIV status and fear of discrimination.

Internal and external stigma are inevitably linked as experienced stigma and discrimination may be internalized and this in turn can have an effect on how PLHIV perceive themselves and their disease. Such a dynamic between internal and external stigma ultimately impacts personal and social relationships and health seeking behavior [25]. Internal stigma has been associated with psychosocial factors, such as depression, low self-esteem, social isolation, and psychological distress [26–29]. Behavioral effects of internal stigma have also been reported, including delay in seeking healthcare, poor treatment adherence, and overall poor physical health [27, 30–33].

Given that the HIV epidemic in Morocco is largely concentrated among key populations who already face social exclusion, and that this may further be compounded and reinforced by HIV-related stigma, it is crucial to evaluate and address stigma to improve the quality of life of PLHIV and facilitate their entry and retention in the care continuum. Although there is a growing body of literature documenting and exploring stigma among PLHIV, there is a paucity of data from Morocco. The present study measures and identifies factors associated with an internal stigma score among PLHIV in Morocco, which may be used to inform the development of effective strategies to reduce stigma and thus improve health outcomes and overall well-being for PLHIV.

## Methods

*The People Living with HIV Stigma Index* was used to collect data for this study. The Stigma Index is an international initiative launched in 2004 by various organizations (Global Network of People Living with HIV, International Community of Women living with HIV/AIDS, UNAIDS) to document the various experiences of PLHIV related to stigma and discrimination. The PLHIV Stigma index (http://www.stigmaindex.org/) is one among many validated instruments used to collect evidence of stigma and discrimination among PLHIV, to advocate for the human rights of PLHIV and to inform the development of effective strategies to reduce stigma experienced among key populations [34]. The overall objective of implementing the Stigma Index in Morocco was to have a more precise measure of the stigma and discrimination experienced by PLHIV in Morocco. Specific objectives included exploration of internal stigma. The study, which received ethics approval from the Casablanca Biomedical Research Ethics Committee, was conducted between September and October 2016. Although a qualitative study was also conducted within the context of this project, the present study focuses on the quantitative study data.

### Study preparation

A steering committee (SC), chaired by the national AIDS program and UNAIDS-Morocco, was commissioned to recruit the research team, validate the study design, review the results, provide recommendations and suggest interventions to address HIV-related stigma based on study results. The Research Department of Association de Lutte Contre le Sida (ALCS), the first NGO operating in HIV/AIDS in Morocco and in the MENA region, and founding member of Coalition PLUS, was selected by the SC to conduct the survey. A 3-day workshop took place for the development of a training module on stigma, the adaptation of the Stigma Index protocol to the local context and the management of ethical aspects. Subsequently, 15 interviewers living with HIV and 5 supervisors were selected and trained over 2 days by ALCS’s team to administer the questionnaire. Interviewers and supervisors were recruited via organizations known for their implication and work with PLHIV and selected by a recruitment committee.

Ethical approval was received by the Casablanca Biomedical Research Ethics Committee (IRB00002504) in the he Faculty of Medicine and Pharmacy of Casablanca at the Hassan II university in Casablanca. Informed written consent was obtained from all participants.

### Sample size and site selection

Inclusion criteria to participate in the study were: being a PLHIV, being 18 years or older, being followed in a center that provides care for PLHIV (regardless of treatment) and providing consent to participate in the study. A cluster sampling method was used to recruit participants in medical care centers across 8 cities in Morocco. A study sample size of 640 was determined (see supplementary information for details regarding sample size calculation and distribution per study site) and a total of 9 sites were selected to recruit participants (Casablanca, Agadir, Rabat, Marrakech (2 sites), Fez, Tangier, Nador and Beni Mellal). These sites were specifically selected to obtain a nationally representative sample of PLHIV. A randomly drawn cluster of 10 PLHIV, consisting of 5 men and 5 women, was drawn at each participating medical care center among the outpatients scheduled for a visit on that specific day. In the event that one or more of the people selected refused to participate, another draw was conducted to complete the cluster.

### Measures

The People Living with HIV Stigma Index questionnaire was adapted and translated into French and Moroccan Arabic dialect “darija”, then tested through a dozen interviews with PLHIV in Casablanca.

### Dependent variable

Internal stigma was evaluated in the questionnaire with the following question: “Over the last 12 months have you felt one of the following feelings due to your serological status”. The seven feelings or beliefs were as follows: I feel ashamed, I feel guilty, I blame myself, I blame others, I have low self-esteem, I feel that I should be punished, and I want to commit suicide. We created an internal stigma score with 1 point added for each of these items.

The internal stigma score was compiled as a score ranging from 0 to 7 to take account increasing levels of internal stigma, wherein a higher score indicated higher levels of internal stigma.

#### Independent variables

*The People Living with HIV Stigma Index* questionnaire collected information across several themes including: sociodemographic characteristics (sex, age, highest level of formal education completed, employment status, job loss in the last 12 months, average household income in the last 12 months, relationship status, belonging to at least one key population (MSM, homosexual or lesbian, trans, sex worker or injecting drug user)), number of years living with HIV, experiences with stigma and discrimination (the reactions of other adult family members when they first knew about HIV status, experience of psychological pressure or manipulation in which HIV-positive status was used by spouse or partner in the last 12 months, avoiding HIV prevention, testing or treatment services for fear of stigmatization by staff, being denied health services (including dental care) because of HIV status, having confronted, challenged or educated someone who was stigmatizing and/or discriminating [the participant] and perceived health status.

Effects or consequences of internal stigma were evaluated based upon 10 affirmations to the following question: “Over the last 12 months, have you done one of the following due to your serological status?”. The affirmations were as follows: I chose not to go to social gatherings, I isolated myself from my family and/or my friends, I decided to stop working, I decided to not ask for work or a promotion, I quit school/training or I did not take advantage of an educational/training opportunity, I decided not to get married, I decided not to have sexual relations, I decided to not have (more) children, I have avoided going to a local health center when I needed, and I have avoided to go to the hospital when I needed.

### Data analysis

Continuous variables were reported as median with IQR and categorical variables as frequencies. Negative binomial regression models with the estimation of Incidence Rate Ratios (IRR) were used to identify characteristics associated with the internal stigma score. Variables with a *p*-value lower than 0.25 in the bivariable analysis were considered eligible to enter the multivariable model. A backward procedure based on the Likelihood Ratio Chi-2 test was used to select variables for the final model. The final model selection was based on statistical significance (*p* < 0.05) and/or pertinence of the variables.

Data analysis was carried out using Stata/SE 14.0 software (StataCorp LP, College Station, USA).

## Results

A total of 626 PLHIV were recruited to the study however 2 trans individuals were excluded from the analysis due to the low number in comparison to male and female participants and thus the inability to analyze as a separate group. This analysis was based on 604 PLHIV who had complete data concerning the internal stigma score.

### Characteristics of participants

More than half (51.3%) of participants were male and median age was 36 [IQR: 28–43] years (Table 1). One quarter (25.3%) had no formal education, close to half (49.8%) were unemployed, and median household income per month was 2000 MAD [1000–3000] (or 220 US dollars) while the minimum wage was 2600 MAD (or 280 US dollars) at the time of the study. Half (50.2%) of the respondents were single, divorced or separated. One third (33.3%) belonged to at least one key population and median years living with HIV was 4 [2–7]. Very discriminatory or discriminatory reactions of other adult family members when they first knew about HIV status was reported among 16.1% of participants. Experiencing (a few times or often) psychological pressure or manipulation by husband, wife or partner in which HIV-positive status was used was reported by 8.2%. More than a third (36.1%) avoided HIV prevention, testing or treatment services for fear of stigmatization by staff, 28.6% reported being denied health services (including dental care) because of HIV status and 39.2% reported having confronted, challenged or educated someone who was stigmatizing and/or discriminating them. Half of the participants (50.3%) perceived their current health status to be fair or poor.

**Table 1:** Characteristics of participants and factors associated with internal stigma score using bivariable negative binomial regression models (N = 604)

	N(%) or median[IQR]	IRR[95%CI]	p-value
*Sex*			
Male	310 (51.3)	1	
Female	294 (48.7)	1.05 [0.95;1.16]	0.308
Age (years)	36 [28–43]	0.998 [0.993;1.003]	0.346
*Highest level of formal education completed*			
No formal education	153 (25.3)	**1.24 [1.04;1.47]**	**0.015**
Primary or secondary school	374 (61.9)	1.15 [0.98;1.35]	0.078
Technical college or University	77 (12.8)	1	
*Current employment status*			
Full-time	141 (23.4)	1	
Part-time	161 (26.8)	0.87 [0.76;1.01]	0.060
Unemployed and not working at all	300 (49.8)	1.02 [0.90;1.15]	0.774
Lost a job*			
Never or once	198 (33.6)	1	
A few times or often	91 (15.5)	**1.34 [1.15;1.55]**	**< 0.001**
Not applicable	300 (50.9)	**1.22 [1.09;1.36]**	**0.001**
Household income in MAD per month (per one thousand unit increase)	2000 [1000–3000]	**0.976 [0.957;0.995]**	**0.013**
*Current relationship status*			
Married or cohabiting	215 (35.8)	1	
In a relationship but not living together	27 (4.5)	1.09 [0.85;1.39]	0.484
Single, divorced or separated	302 (50.2)	**1.13 [1.02;1.26]**	**0.023**
Widow or widower	57 (9.5)	1.01 [0.84;1.21]	0.947
*Belongs to at least one key population*			
No	400 (66.7)	1	
Yes	200 (33.3)	**1.20 [1.08;1.33]**	**< 0.001**
Number of years living with HIV	4 [2–7]	0.999 [0.989;1.010]	0.896
Reactions of other adult family members when they first knew about HIV status*			
Very discriminatory or discriminatory	96 (16.1)	**1.41 [1.22;1.63]**	**< 0.001**
No difference	95 (15.9)	1.14 [0.98;1.33]	0.094
Very supportive or supportive	169 (28.4)	1	
Not Applicable	236 (39.6)	1.07 [0.95;1.21]	0.283
Psychological pressure or manipulation by husband or wife or partner in which HIV-positive status was used*			
Never or once	495 (91.8)	1	
A few times or often	44 (8.2)	**1.43 [1.21;1.70]**	**< 0.001**
*Avoiding HIV prevention / testing / treatment services for fear of stigmatization by staff*			
No	364 (63.9)	1	
Yes	206 (36.1)	**1.21 [1.10;1.34]**	**< 0.001**
Denied health services (including dental care) because of HIV status*			
No	238 (39.7)	1	
Yes	171 (28.6)	**1.35 [1.20;1.51]**	**< 0.001**
Not applicable	190 (31.7)	1.0 [0.96;1.21]	0.216
Confronted, challenged or educated someone who was stigmatizing and/or discriminating *†			
*No*	364 (60.8)	1	
*Yes*	235 (39.2)	**1.28 [1.16;1.41]**	**< 0.001**
*General perceived health status (current)*			
Excellent or very good or good	297 (49.7)	1	
Fair or poor	300 (50.3)	**1.33 [1.21;1.47]**	**< 0.001**

*In the last 12 months; † stigma and discrimination against the participant

### Internal stigma

**Fig. 1:**
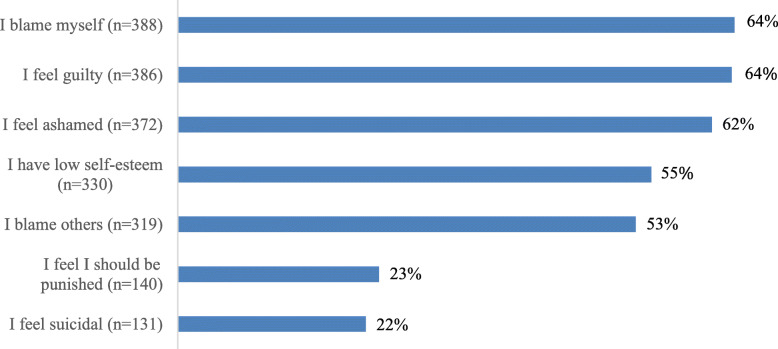
Internal stigma: Frequency of beliefs and feelings (*N* = 604)

Among the 7 negative feelings or beliefs of internal stigma, 64% of respondents reported blaming themselves and feeling guilty and 62% of respondents reported feeling ashamed (Fig. 1). More than half reported having low self-esteem and blaming others. Less than a quarter had experiences of feeling that they should be punished and feeling suicidal.

Median [IQR] internal stigma score was 4 [2–5]. Seventy-one respondents (11.8%) reported no indications of internal stigma. Among the 88.2% that reported indications of internal stigma, 4.8% were concerned by all the 7 items of internal stigma, with a score equal to 7 (Table 2).

**Table 2:** Descriptive of internal stigma score (N = 604)

	N	%	Cumulated %
0	71	**11.8**	**11.8**
1	58	**9.6**	**21.4**
2	71	**11.8**	**33.2**
3	90	**14.9**	**48.1**
4	101	**16.7**	**64.8**
5	115	**19.0**	**83.8**
6	69	**11.4**	**95.2**
7	29	**4.8**	**100.0**
Total	604	**100.0**	

### Internal stigma effects or consequences

**Fig. 2:**
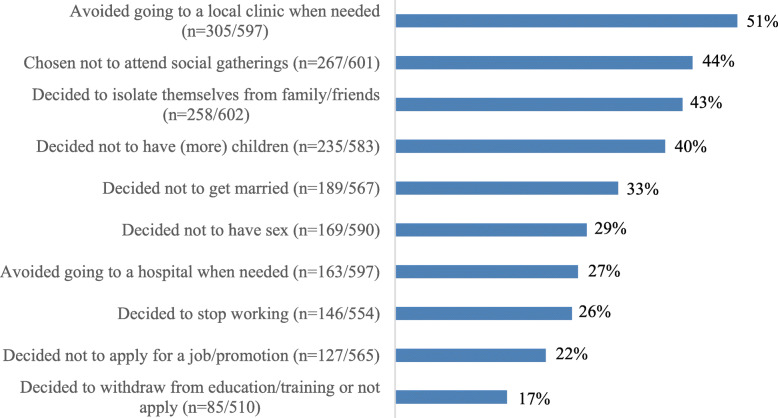
Internal stigma: Frequency of effects or consequences (N = 604)

More than half (51%) of the respondents avoided going to the local clinic when they needed and 27% avoided going to the hospital (Fig. 2). Forty-four percent chose not to attend social gatherings and 43% decided to isolate themselves from family and/or friends. The decision to not have (more) children was reported by 40% of respondents, the decision to not to get married and not to have sex were reported by 33 and 29%, respectively. Slightly more than a quarter decided to stop working, 22% did not apply for a job/promotion and 17% withdrew from education/training or decided not to apply due to internal stigma.

### Factors associated with internal stigma score

#### Bivariable analysis

In the bivariable analysis, having no formal education, having lost a job a few times or often and not being in a position to lose a job in the last 12 months, being single, divorced or separated, and belonging to at least one key population were predictive of an increase of the internal stigma score (Table 1). Having very discriminatory or discriminatory reactions of other adult family members when they first knew about HIV status and being a few times or often under psychological pressure or manipulation by husband, wife or partner in which HIV-positive status was used were associated with an increase of the internal stigma score. Avoiding HIV prevention / testing / treatment services for fear of stigmatization by staff, being denied health services because of HIV status, and having confronted, challenged or educated someone who was stigmatizing and/or discriminating, and reporting current general health status as fair or poor were also predictive of an increase of the internal stigma score.

In contrast, a higher household income per month was predictive of a decrease of the internal stigma score.

No significant association was detected between sex, age, current employment status, number of years living with HIV, and the internal stigma score.

#### Multivariable analysis

The final multivariable model included 495 respondents; results are presented in Table 3. It was adjusted for current relationship status (being single or divorced or separated) and for being a few times or often under psychological pressure or manipulation by husband or wife or partner in which HIV-positive status was used. In the multivariable analysis, not having a formal education compared to finishing technical college or university was independently associated with an increase in the internal stigma score. Belonging to at least one key population, having very discriminatory or discriminatory reactions of other adult family members when they first knew about HIV status, avoiding HIV prevention / testing / treatment services for fear of stigmatization by staff and being denied health services because of HIV status and reporting fair or poor perceived health, were also significantly associated with an increase of the internal stigma score.

**Table 3:** Factors independently associated with internal stigma score using multivariable negative binomial regression (*N* = 495)

	aIRR[95%CI]	p-value
*Highest level of formal education completed*		
No formal education	**1.38 [1.16;1.65]**	**< 0.001**
Primary or secondary school	1.15 [0.98;1.35]	0.082
Technical college or University	1	
*Current employment status*		
In full-time	1	
In part-time	**0.84 [0.73;0.96]**	**0.013**
Unemployed and not working at all	0.92 [0.82;1.04]	0.183
*Current relationship status*		
Married or cohabiting	1	
In a relationship but not living together	1.12 [0.88;1.43]	0.352
Single or divorced or separated	1.11 [0.99;1.24]	0.074
Widow or widower	0.99 [0.83;1.19]	0.952
*Belongs to at least one key population*		
No	1	
Yes	**1.15 [1.03;1.28]**	**0.016**
Reactions of other adult family members when they first knew about HIV status*		
Very discriminatory or discriminatory	**1.28 [1.11;1.49]**	**0.001**
No difference	1.05 [0.90;1.23]	0.501
Very supportive or supportive	1	
Not applicable	1.09 [0.96;1.23]	0.188
Psychological pressure or manipulation by husband or wife or partner in which HIV-positive status was used*		
Never or once	1	
A few times or often	1.17 [1.00;1.37]	0.053
*Avoiding HIV prevention / testing / treatment services for fear of stigmatization by staff*		
No	1	
Yes	**1.16 [1.05;1.28]**	**0.004**
Denied health services (including dental care) because of HIV status*		
No	1	
Yes	**1.16 [1.03;1.32]**	**0.014**
Not applicable	1.05 [0.93;1.18]	0.441
*Perceived health*		
Excellent or very good or good	1	
Fair or poor	**1.23 [1.11;1.36]**	**< 0.001**

*In the last 12 months

Having part-time employment compared to full time employment was significantly associated with a decrease in the internal stigma score.

## Discussion

This study provides unique insight into experienced internal stigma, and its effects and/or consequences, among PLHIV in Morocco and supports available data on internal stigma at the regional [7, 35–37] and international levels [26, 38, 39]. Among this representative sample of PLHIV, 88.2% reported experiencing internal stigma. Factors at the individual, social and structural levels were associated with higher internal stigma scores. Furthermore, we found that the HIV-related internal stigma among PLHIV impacted certain personal and professional life decisions, participation in social activities, and healthcare seeking. This study further highlights the need for multi-level interventions to address the internal stigma experienced by PLHIV in Morocco and elsewhere [1, 40, 41].

### Study population

The temporospatial cluster sampling used in this study allowed us to gather a representative sample of PLHIV in Morocco; the 8 care centers that were chosen to recruit participants are known to be the most solicited by PLHIV in Morocco. The average age and the relationship situation of the study participants are very similar to those seen among the cohort of PLHIV followed in the care centers in Morocco where average age is 38.1 years and 34.0% are single, 41.4% are married and 9.3% are widowed [42]. The study sample confirms the young character of the PLHIV population in Morocco. The median age of participants was 36 years and PLHIV under 40 represent more than 65% of participants. These results are in accordance with the national statistics published in 2015 [42] in which the reported proportion of young adults (between 25 and 44 years) infected with HIV was 65%. More than half of the study participants were not in a relationship at the time of the interview (either single, divorced or separated or widowed) and the rate of widowed women was significantly higher than for men (18% vs 2%), as found in another study [43]. The level of education, employment status and economic level reflect the precarious and vulnerable situation of PLHIV and key populations in general [44].

### Prevalence of internal stigma, internal stigma score, and effects or consequences of internal stigma

Our results show that HIV-related internal stigma is predominant (88.2%) among PLHIV; blaming oneself, feeling guilty, and feeling ashamed were the most frequently reported indications. Blaming oneself has also been reported in other studies within the region: 70% in Egypt [36], 49% in Algeria [37] and 46% in Yemen [35]. This is also observed in other regions of Africa: 49.2% in South Africa [38] and 40% in Cameroon [39]. However, the proportion of PLHIV reporting low self-esteem in Morocco (55%) and in Egypt (60%) is much higher compared to Algerian PLHIV (17%).

More than half of the participants (51.9%) reported 4 or more indications of internal stigma. We note that there is variability in the level of experienced internal stigma, including those who report no indications [26, 45]. Additionally, internal stigma impacted certain decisions that touched various spheres of life including personal and social relationships (not getting married, not having sex, not going to social gatherings, social isolation), career (not applying for employment/training, withdrawing or not applying for employment/training) and health (avoiding to seek care when needed). Several studies have shown an association between internal stigma and poor affective health and well-being as well as poor health care behaviors [25, 27, 46].

Certain sociodemographic factors, such as not having formal education, were associated with a higher level of internal stigma score, as reported elsewhere [47]. The fact that part-time employment, compared to full-time employment, was associated with a lower level of internal stigma score may reflect a potential protective effect of limiting exposure to HIV-related stigma that has been documented in the workplace [48]. The negative impact of stigma on education and professional evolution which concerned 16–26% of the participants further supports these results. No association was detected between age or gender, in contrast to other studies [38, 46, 49, 50]. We note, however, that women were significantly more likely to declare six or more negative feelings or beliefs of internal stigma compared to men (*p* = 0.013). As current relationship status was statistically significant in the bivariable analysis, we chose to adjust for this variable in the final model given research that has shown a positive association between stigma and living with spouse [51], the potential social consequences of HIV-related stigma on the family [52] and the importance of familial support [53].

Belonging to at least one key population was also significantly associated with an increase of the internal stigma score, possibly due to experiences of intersectional stigma related to serological status and to judgement by others for “immoral and unacceptable acts” [20, 54]. Indeed, commercial sex work and same-sex relationships are condemned by the society and the law and can contribute to self-blame among key populations [55].

In the past decade there has been an increasing focus on understanding “intersectional stigma” which encompasses the phenomenon that an individual or a group can experience stigma related to various social categories (or more appropriately social inequalities), and in a cumulative manner [21, 56]. It is thus necessary for interventions to have a holistic approach, taking into account intersectional stigma, to have maximal effect on reducing social and health inequalities experienced by PLHIV [21, 57, 58].

Respondents who reported very discriminatory or discriminatory reactions from other adult family members upon learning of serostatus had higher internal stigma scores. Greater internalized stigma has also been associated with lower HIV status disclosure to family members [59]. Furthermore, there is evidence that serostatus disclosure may be particularly difficult among individuals who consider religion an important aspect of their lives due to fear of stigma and discrimination [60]. Lack of disclosure among family members (and others), however, will also have an impact on potential support and lead to social isolation [25, 61]. Experience of psychological pressure or manipulation by spouse or partner in which HIV status was used was not significant (*p* = 0.053) but was kept in the final model, much for the same reasons as relationship status, as it reflects the complex role of family in HIV-related stigma experienced by PLHIV.

More than a quarter (28.6%) of the study participants reported being denied health services and this was significantly associated with the internal stigma score. Although this concerned a non-negligible proportion of PLHIV in Morocco, this is low compared to 53% reported in Algeria and in Egypt [36, 37]. Perceived or enacted stigma has been identified as a major barrier to accessing healthcare services and treatment [24, 30, 50, 62, 63]. Our results show that 52% of participants decided not to visit a local clinic even when it was necessary. This result is higher than proportions reported by other countries such as Cameroon (8%) or Democratic Republic of the Congo (18%), but it is similar to other countries in the region, such as Algeria (48%) and Yemen (33%) [35, 37]. Avoiding HIV prevention / testing / treatment services for fear of stigmatization by staff was associated with a higher internal stigma score, which supports findings from other studies. HIV-related stigma can work through various mechanisms to impact engagement in HIV-related care such as ART adherence and care retention [29]. Avoiding healthcare services has an obvious impact on the ability to test and treat PLHIV and on a broader scale likely contributes to challenges in implementing effective HIV strategies in the region [64]. Finally, and more generally, poor health seeking behavior also has a negative impact on overall health and well-being. Previous research found that severe HIV symptoms made PLHIV felt negative about themselves [19, 65]. Indeed, our findings confirm that a fair or poor perceived health status is significantly associated with an increase of the internal stigma score.

### Strengths and limitations

Given the limited data available regarding PLHIV in the MENA region, this study contributes valuable information on internal stigma among a representative sample of PLHIV. In conjunction with other countries in the region that also conducted the Stigma Index, these results further support the prevalence of internal stigma. Use of the Stigma Index to measure internal stigma is also a strength of this study as it is a validated instrument that has been used internationally. Implication of PLHIV in the study, as trained interviewers, is also a strength as they are provided an opportunity to be an active part of the study and their role may also put participants at ease for completion of the questionnaire. One limitation concerns the lack of dedicated questions on stigma related to key population membership, and thus intersectional stigma could not specifically be analyzed; such questions have been added to a newer version of the Stigma Index tool [34]. Additionally, as this is a cross-sectional study, we are unable to evaluate stigma and discrimination over time. As HIV is now a chronic condition, it is increasingly important to collect longitudinal data regarding stigma [22]. Finally, due to low participation, we were unable to analyze information regarding trans participants. More visibility to this population, which is disproportionately affected by HIV globally [66] and who also suffer from criminalization for their sexual orientation and gender identity in the region, is urgently needed.

## Conclusions

Although Morocco is recognized for its national response to the HIV epidemic [67], the present analysis suggests that the national response must also urgently address HIV-related stigma that is prevalent among PLHIV, to improve linkage and retention in care, and further accelerate efforts to end the HIV epidemic in this country and meet UNAIDS 90–90-90 goals [64]. Interventions cannot be limited to the individual level [40, 41], but must also target community, structural and policy levels. Indeed, multi-level interventions are necessary to address the social and structural inequalities that create and reinforce stigma and discrimination experienced by PLHIV [1]. This framework should guide future research studies. Community-based research may be particularly adapted for further exploring ways to address HIV-related stigma, given the inherent implication of stakeholders at all levels (communities, healthworkers, policymakers, researchers, etc).

Empowerment and mobilization of PLHIV and communities has been a driving force in the history of the HIV epidemic, and their role in drawing attention to and implementing strategies to reduce HIV-related and intersectional stigma cannot be overlooked. The results presented in this study, and others, should be used to inform culturally adapted interventions, programs and political policies with the larger aim of changing the economic, social and political environments to protect the rights and improve the quality of life of PLHIV and key populations.

## Supplementary Information

**Additional file 1.**

## Data Availability

The data that support the findings of this study are available from the corresponding author upon reasonable request.

## Abbreviations

aIRR: Adjusted Incidence Rate Ratio
ALCS: Association de Lutte Contre le Sida
CI: Confidence Interval
IQR: Inter-Quartile Range
MAD: Dirham Marocain
MENA: Middle East and North African
MSM: Men who have Sex with Men
PLHIV: The People Living with HIV
