# Supplementary material for: Determinants and effects or consequences of internal HIV-related stigma among people living with HIV in Morocco
Source: BMC Public Health. 2021 Jan 19;21:163. doi: 10.1186/s12889-021-10204-1 (PMC7815182; doi:10.1186/s12889-021-10204-1)
Supplement: Supplementary file 1 — Additional file 1. [file 12889_2021_10204_MOESM1_ESM.docx]

**SUPPLEMENTARY INFORMATION**

*Sample size calculation*

The calculation of the sample size was based on the results of a study conducted in Morocco in 2006 among 133 PLHIV which reported that nearly 40% of participants had been discriminated in a health care setting. To detect a 10% variation, with a study power of 80%, a 95% confidence level of a one-sided test and a design effect of 2, the sample size is 602 people rounded up to 640.

Formula used to calculate the sample size


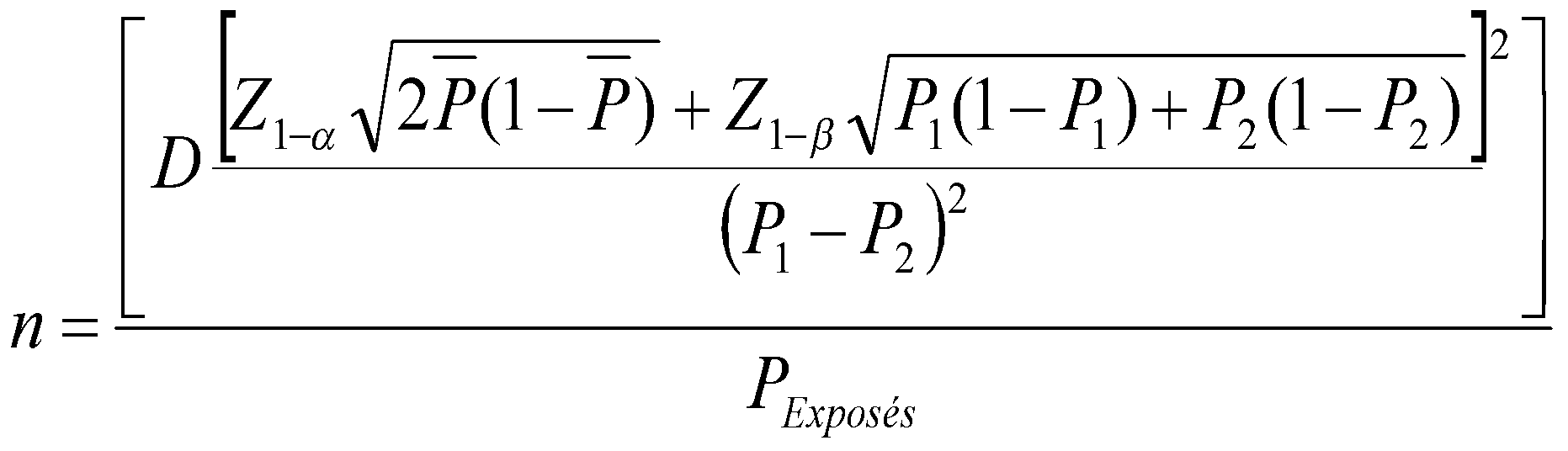


Exposed

D = Effect of the sampling plan

P1 = Initial percentage.

P2 = Percentage target (the difference P2 - P1 is the span of the variation that one wants to be able to detect)

Calculation of the sample size:

For D = 2, P1 = 40% , P2 = 50% , PExposed = 100%, Z1-α = 1.645, Z1-β = 0.83

*Sample size per site*

The sample size contribution of each of the sites selected to participate in the study was calculated based on the number of PLHIV followed per site (Table 4):

**Table 4: Distribution of the sample by selected medical care centers**

| **N° center** | **Medical care centers** | **Adults on ART** | **% in the national active file population** | **Sample size** |
| --- | --- | --- | --- | --- |
| 1 | Casablanca | 2236 | 28.0% | 180 |
| 2 | Agadir | 3002 | 37.5% | 240 |
| 3 | Rabat | 676 | 8.5% | 50 |
| 4 | Marrakech (Site 1) | 943 | 11.8% | 70 |
| 5 | Marrakech (Site 2) | 448 | 5.6% | 40 |
| 6 | Fès | 160 | 2.0% | 10 |
| 7 | Meknès | 133 | 1.7% | 10 |
| 8 | Tanger | 338 | 4.2% | 30 |
| 9 | Nador | 64 | 0.8% | 10 |
| Total | | 8000 | 100.0% | 640 |
